# Clonal anergy of CD117^+^chB6^+^ B cell progenitors induced by avian leukosis virus subgroup J is associated with immunological tolerance

**DOI:** 10.1186/s12977-018-0463-9

**Published:** 2019-01-03

**Authors:** Shuhai He, Gaoying Zheng, Defang Zhou, Gen Li, Mingjun Zhu, Xusheng Du, Jing Zhou, Ziqiang Cheng

**Affiliations:** 1 College of Veterinary Medicine, Shandong Agricultural University, Tai’an, 271018 China; 2 College of Husbandry and Veterinary, Xinyang Agriculture and Forestry University, Xinyang, 464000 China

**Keywords:** Avian leukosis virus subgroup J, B cell progenitor, B cell anergy, Immunological tolerance, Congenital infection

## Abstract

**Background:**

The pathogenesis of immunological tolerance caused by avian leukosis virus subgroup J (ALV-J), an oncogenic retrovirus, is largely unknown.

**Results:**

In this study, the development, differentiation, and immunological capability of B cells and their progenitors infected with ALV-J were studied both morphologically and functionally by using a model of ALV-J congenital infection. Compared with posthatch infection, congenital infection of ALV-J resulted in severe immunological tolerance, which was identified as the absence of detectable specific antivirus antibodies. In congenitally infected chickens, immune organs, particularly the bursa of Fabricius, were poorly developed. Moreover, IgM-and IgG-positive cells and total immunoglobulin levels were significantly decreased in these chickens. Large numbers of bursa follicles with no differentiation into cortex and medulla indicated that B cell development was arrested at the early stage. Flow cytometry analysis further confirmed that ALV-J blocked the differentiation of CD117^+^chB6^+^ B cell progenitors in the bursa of Fabricius. Furthermore, both the humoral immunity and the immunological capability of B cells and their progenitors were significantly suppressed, as assessed by (a) the antibody titres against sheep red blood cells and the Marek’s disease virus attenuated serotype 1 vaccine; (b) the proliferative response of B cells against thymus-independent antigen lipopolysaccharide (LPS) in the spleen germinal centres; and (c) the capacities for proliferation, differentiation and immunoglobulin gene class-switch recombination of B cell progenitors in response to LPS and interleukin-4(IL-4) in vitro.

**Conclusions:**

These findings suggested that the anergy of B cells in congenitally infected chickens is caused by the developmental arrest and dysfunction of B cell progenitors, which is an important factor for the immunological tolerance induced by ALV-J.

## Introduction

Avian leukosis virus subgroup J (ALV-J), an oncogenic retrovirus, causes myeloid leukosis and various other neoplastic diseases in both broiler and layer chickens [1, 2]. In addition to causing neoplastic diseases and reducing production performance, the serious effect of ALV-J on birds is immunosuppression [3, 4]. Like other exogenous avian leukosis viruses, ALV-J can be transmitted in vertical or horizontal infection. Generally, chickens infected by the congenital transmission of ALV-J are prone to immunological tolerance. Congenitally infected chickens are characterized by the presence of high levels of virus in the blood and tissues, but the absence of antivirus-specific antibodies [5–7]. In particular, immunological tolerance induced by ALV-J is an essential factor for neoplasia and opportunistic infection [8–10]. However, little is still known about the pathogenesis of immunological tolerance caused by the congenital infection of ALV-J. Previous studies have suggested the presence of lymphocyte depletion in special areas of immune organs and the unusual expression of cytokine genes associated with immunity in chickens that are inoculated with ALV-J after hatching [11–13]. These data indicated that ALV-J has selective effects on lymphocyte type and development stage.

Immunological tolerance is a state of non-response or low-response of B or T cells to a specific antigen. Abnormal development and dysfunction of immune cells infected with virus are also among the causes of immunological tolerance [14]. B cells play an important role in antiviral humoral immunity. However, some viruses, such as influenza virus, can induce B cell anergy [15]. In this state, anergic B cells fail to complete differentiation, to proliferate, and to make antibodies [16, 17]. Experimental data collected in animal models and humans have also shown that the B cell anergy induced by hepatitis B virus (HBV) and human immunodeficiency virus (HIV) can cause immunological tolerance, especially in the context of congenital infection [18, 19]. Studies in our lab and others have shown that ALV-J has tissue tropism in the lymphocytes of the bursa of Fabricius [20, 21]. ALV-J can alter the expression of genes associated with growth regulation, immune system processes, and neoplasia regulation in bursal cells [22, 23]. Importantly, the bursa of Fabricius, unique to birds, is where B cell differentiation and maturation are induced. B cell precursors gradually develop after colonizing the bursal epithelium and migrate to secondary lymphoid organs after maturation to participate in acquired immunity [24, 25]. These results motivated us to investigate the pathogenesis of immunological tolerance induced by ALV-J from the perspective of whether the virus affects B cell development and function.

Chickens congenitally infected with ALV-J were more prone to immunological tolerance than those horizontally infected, which suggested the possibility that ALV-J might affect early B cell development. Indeed, whether the pro-B cell is normal will determine B cell development and function, such as the development of the bursal follicles, the rearrangement of the antigen receptor gene fragments, and the immunoglobulin (Ig) gene class-switch recombination (CSR) [26, 27]. In the present study, the development, differentiation, and immunological capability of B cells and their progenitors infected with ALV-J were studied both morphologically and functionally in both in vivo and in vitro experiments.

## Results

### Chickens infected at ED 6 suffered immunological tolerance and showed development arrest of bursal follicles and B cells

**Fig. 1:**
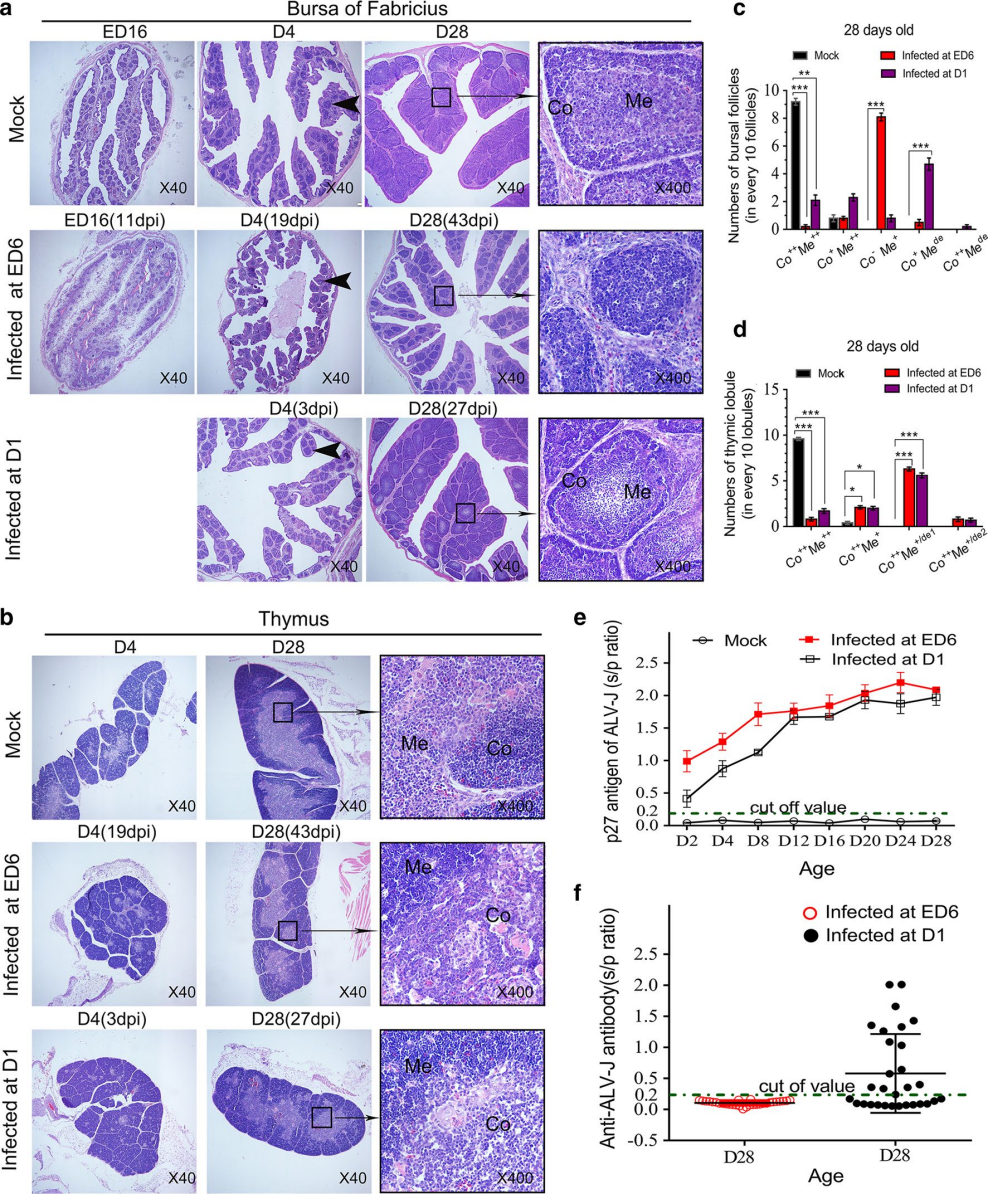
Congenital infection of ALV-J caused abnormal development of immune organs and induced immunological tolerance. **a** Compared with the posthatch infection of chickens (n = 30) and mock infection of chickens (n = 30), ED6 infection of chickens (n = 30) was characterized by (1) poorly developed bursas of Fabricius, with bursal follicles with no differentiation into medulla (Me) and cortex (Co); (2) markedly decreased numbers of bursal follicles; and (3) hyperplastic interstitial connective tissue. **b** In chickens infected at ED 6 and infected at D 1, mild depletion of lymphocytes and multiplication of reticular cells were evident in the medulla. Tissue sections were stained by H&E (magnification: ×40, ×400). Arrows indicate the bursal follicles. **c**, **d** Quantitation of the degree of lesion. “++” indicates good development, “+”indicates dysplasia, “de” indicates depletion of lymphocytes, “de1” indicates mild depletion of lymphocytes, “de2” indicates severe depletion of lymphocytes. **p < 0.01, ***p < 0.001, two-way ANOVA was performed. **e** and **f** Compared with chickens infected at D 1 (n = 30), chickens (n = 30) infected at ED 6 showed severe immunological tolerance, which was characterized by the presence of high levels of ALV-J specific p27 antigen but the absence of detectable anti-ALV-J antibodies. Data are expressed as the mean ± SEM

Consistent with previous studies [6, 28, 29], current ELISA test results showed that chickens infected at day 6 of embryogenesis (ED 6) had high levels of specific p27 antigen of ALV-J but no detectable anti-ALV-J antibody in vivo. The anti-ALV-J antibody was detected in a small number of chickens infected at 1 day (D 1) posthatch (Fig. 1e, f). The results suggested that all experimental chickens infected with ALV-J at ED 6 have developed immunological tolerance.

As shown in Fig. 1a, b, histopathologically, the immune organs of chickens infected with ALV-J at ED 6 were poorly developed. Particularly, the bursa of Fabricius was characterized by poorly developed bursal follicles, with no differentiation into cortex and medulla, and the bursa of Fabricius had markedly decreased numbers of bursal follicles separated by hyperplastic interstitial connective tissue (Fig. 1a, c). However, despite the stunting of development and the decrease in lymphocyte numbers in the medulla, the bursal follicles in chickens infected with ALV-J at D 1 could differentiate into medulla and cortex as normal chickens (Fig. 1a, c). In chickens infected at ED 6 and D 1, mild depletion of lymphocytes and multiplication of reticular cells were detected in the medulla (Fig. 1b, d). However, the effect of ALV-J on the thymus was not as serious as that observed on the bursa of Fabricius. Compared with chickens infected posthatch, congenital infection of ALV-J (infected at ED 6) resulted in decreased chB6^+^ B cells in the bursal follicles and spleen (Fig. 3b, c). Collectively, these findings indicated that ALV-J interferes with the development of B cells at the early stage of development in the context of ALV-J congenital infection.

### Chickens infected with ALV-J at ED 6 showed decreased numbers of IgM and IgG cells in immune organs and decreased total immunoglobulin in the blood

**Fig. 2:**
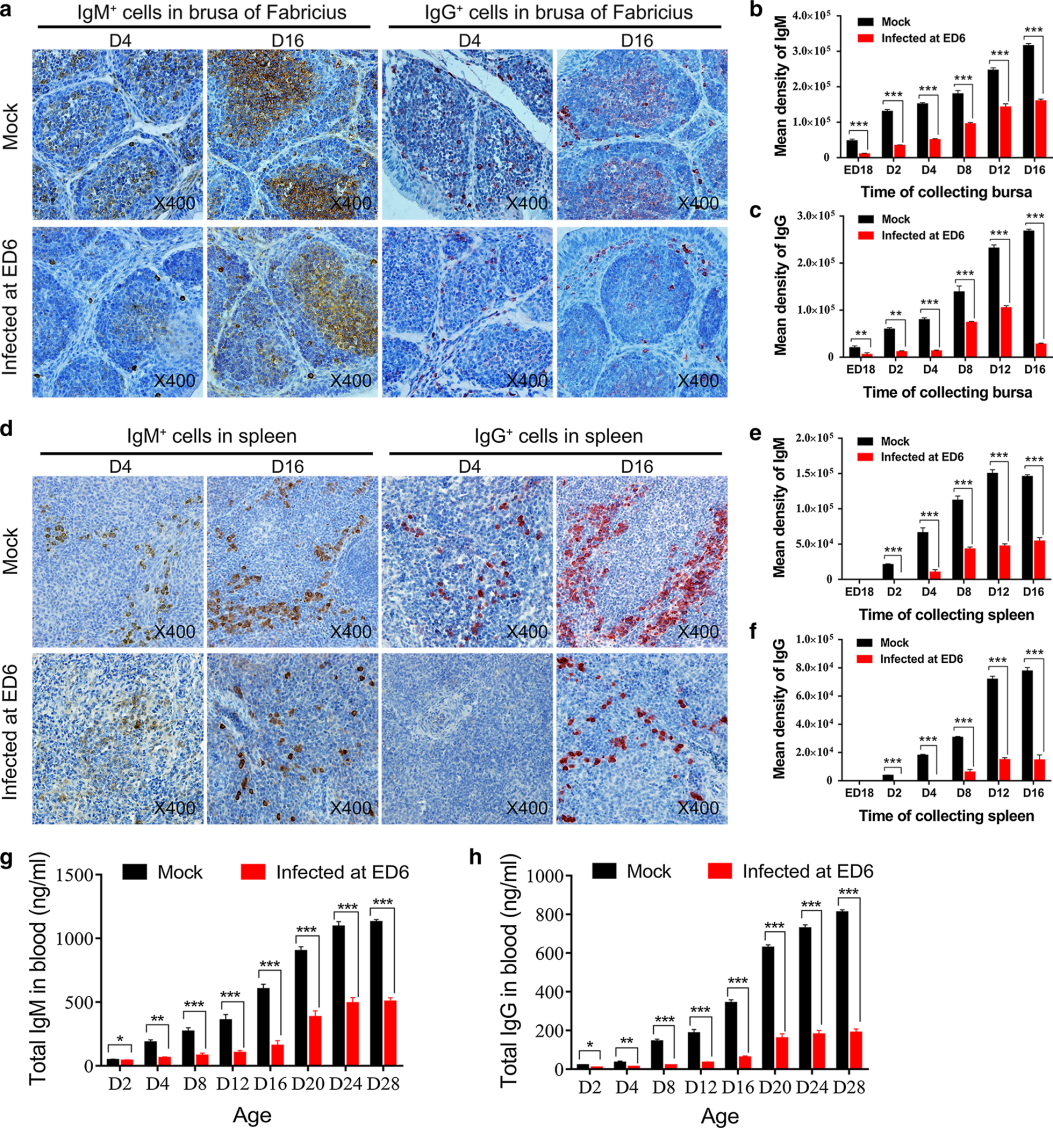
ALV-J inhibited the production of IgM^+^ and IgG^+^ cells in vivo. **a** and **d** Tissue sections of the bursas of Fabricius and spleens obtained from chickens (n = 36) infected at ED 6 or mock-infected chickens (n = 36) were stained by IHC using rabbit anti-IgM/IgG antibody. In these tests, the bursas of Fabricius and spleens were sampled at 6 time points (six samples were collected per time point). In infected chickens, the number of IgM^+^ or IgG^+^ cells in the bursa of Fabricius and spleen were fewer than those in the mock-infected chickens. Sections were stained by DAB (Brown) or AEC (red) and counterstained by haematoxylin (magnification: × 400). Arrow shows the bursal follicles. **b** and **c** Quantitation of IgM- or IgG^−^positive products in the bursa of Fabricius. **e** and **f** Quantitation of IgM- or IgG^−^positive products in the spleen; *p < 0.05, **p < 0.01, ***p < 0.001, two-way ANOVA was performed. **g** and **h** ELISA of total IgM and IgG levels in the blood of infected or control chickens; *p < 0.05, **p < 0.01, ***p < 0.001, two-way ANOVA was performed

To further investigate the effect of ALV-J on the development of immunoglobulin-secreting cells in congenitally infected chickens, the expression of IgM^+^ and IgG^+^ cells in the bursa of Fabricius and spleen in chickens infected at ED 6 and uninfected chickens was detected and compared starting during embryogenesis. Immunohistochemistry (IHC) test results showed that fewer IgM^+^ and IgG^+^ cells presented in the bursas of Fabricius and spleens of infected chickens than those of uninfected chickens (Fig. 2a, d). Starting at the age of ED 18, IgM^+^ and IgG^+^ cells were found in the bursas of Fabricius and spleens of uninfected chickens. The IgM^+^ and IgG^+^ cells in not only the bursas of Fabricius but also the spleens of infected chickens were significantly fewer in number compared with uninfected chickens. IgM^+^ cells were absent from the spleens of infected chickens at D 2. Surprisingly, until the age of D 4, the IgG^+^ cells still had not presented in the spleens of infected chickens. The quantitative analysis results of the IHC-positive products between the infected and uninfected chickens were significantly different (Fig. 2b, c, e, f). The above results indicated that ALV-J affects the capacity of B cells to produce IgM and IgG. Furthermore, the level of total IgM and IgG in the infected chickens was much lower than those in the uninfected chickens (Fig. 2g, h).

### ALV-J blocked the differentiation of CD117^+^chB6^+^ B cell progenitors

**Fig. 3:**
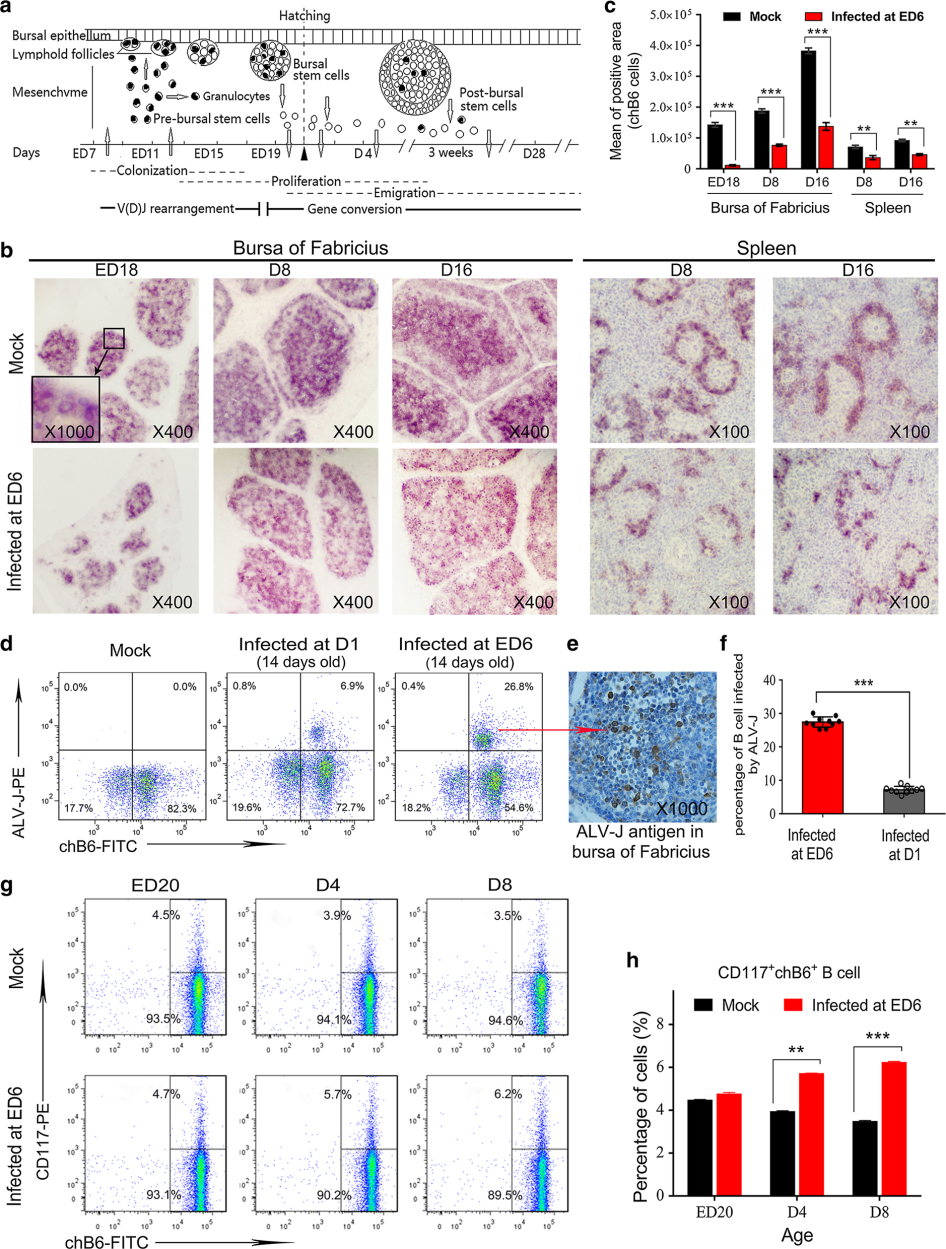
ALV-J blocked the differentiation and maturity of CD117^+^chB6^+^ B cell progenitors. **a** Schematic representation of bursal B cell morphogenesis and gene expression. **b** IHC staining for chB6 antigen in the bursas of Fabricius and spleens of mock-infected chickens and chickens infected at ED 6 using mouse anti-chB6 Ab. Fewer B cell clones (as shown in the inset) were present in the bursal follicles and splenic nodules of infected chickens; the medulla was absent in the bursal follicles of chickens infected at ED 6. Sections are the consecutive sections from those used for histological examination, which were stained by BCIP/NBT (black purple) (magnification: ×400, ×100). **c** Quantitation of **b**; **p < 0.01, ***p < 0.001, two-way ANOVA was performed. **d** Flow cytometry analysis for the differentiation of B cell progenitors. In this test, the mock-infected chickens (n = 15) and chickens infected at ED 6 (n = 15) were used. **e** Quantitation of **d**; **p < 0.01, ***p < 0.001, two-way ANOVA was performed. **f** Flow cytometry analysis for the percentage of ALV-J-infected B cells, which showed that inoculating with ALV-J during the embryonic period can cause more B cells to be infected. In this test, mock-infected chickens (n = 10), chickens infected at ED 6 (n = 10), and chickens infected at D 1 (n = 10) were used. **g** IHC staining for ALV-J antigen in the bursas of Fabricius of chickens infected at ED 6. Sections were stained by DAB (Brown) and counterstained by haematoxylin (magnification: ×1000). **h** Quantitation of **f**; ***p < 0.001, unpaired t-test was performed to determine the statistical difference

As shown in Fig. 3a, B cells in the bursal follicles derived from the haematopoietic stem cells and colonized in the epithelial buds, and the pre-bursal stem cells successively developed in the medulla and cortex around hatching [24, 25]. Most of the B cell proliferation takes place in the cortex, not in the medulla, which mainly consists of nondividing cells [30, 31]. Consistent with the abovementioned histopathology findings, the IHC test results confirmed that the early development of B cells in bursal follicles was seriously affected. The same situation applied to the spleen (Fig. 3b, c). Generally, there were fewer B cells in the spleens of chickens infected with ALV-J at ED 6. Moreover, there were far fewer germinal centres (GCs) in the spleens of these infected chickens.

An interesting finding is that infection of ALV-J during the embryonic period can cause more (approximately 27%) of B cells to be infected, whereas infection with ALV-J after hatching can only cause a small number (approximately 7%) of B cells to be infected (Fig. 3d, f). Consistent with previous studies, which reported that the positive staining for ALV-J antigen was mainly detected in the medulla [32], current IHC results showed that these infected B cells were mainly present in the medulla. These positive cells were larger in size and higher in nuclear to cytoplasm ratio compared with other uninfected bursal lymphocytes (Fig. 3e), which suggested that these infected cells were in the early stage of development.

To further investigate whether ALV-J affected the development and differentiation of B cell progenitors, bursal cells in the chickens infected at ED 6 or the uninfected chickens at different ages were used for flow cytometry analysis. The results showed that, at the ages of ED 20, D 4, and D 8, the ratios of CD117^+^ B cell progenitors to CD117^−^ B cells in the infected chickens were 0.050, 0.061, and 0.073, respectively, but the ratios of CD117^+^ B cell progenitors to CD117^−^ B cells in uninfected chickens were 0.045, 0.040, and 0.036, respectively (Fig. 3g, h). These data suggested that the differentiation and maturity of B cell progenitors were arrested by ALV-J.

### B cell function and responses to antigens and pathogens were impaired in ALV-J congenitally infected chickens

**Fig. 4:**
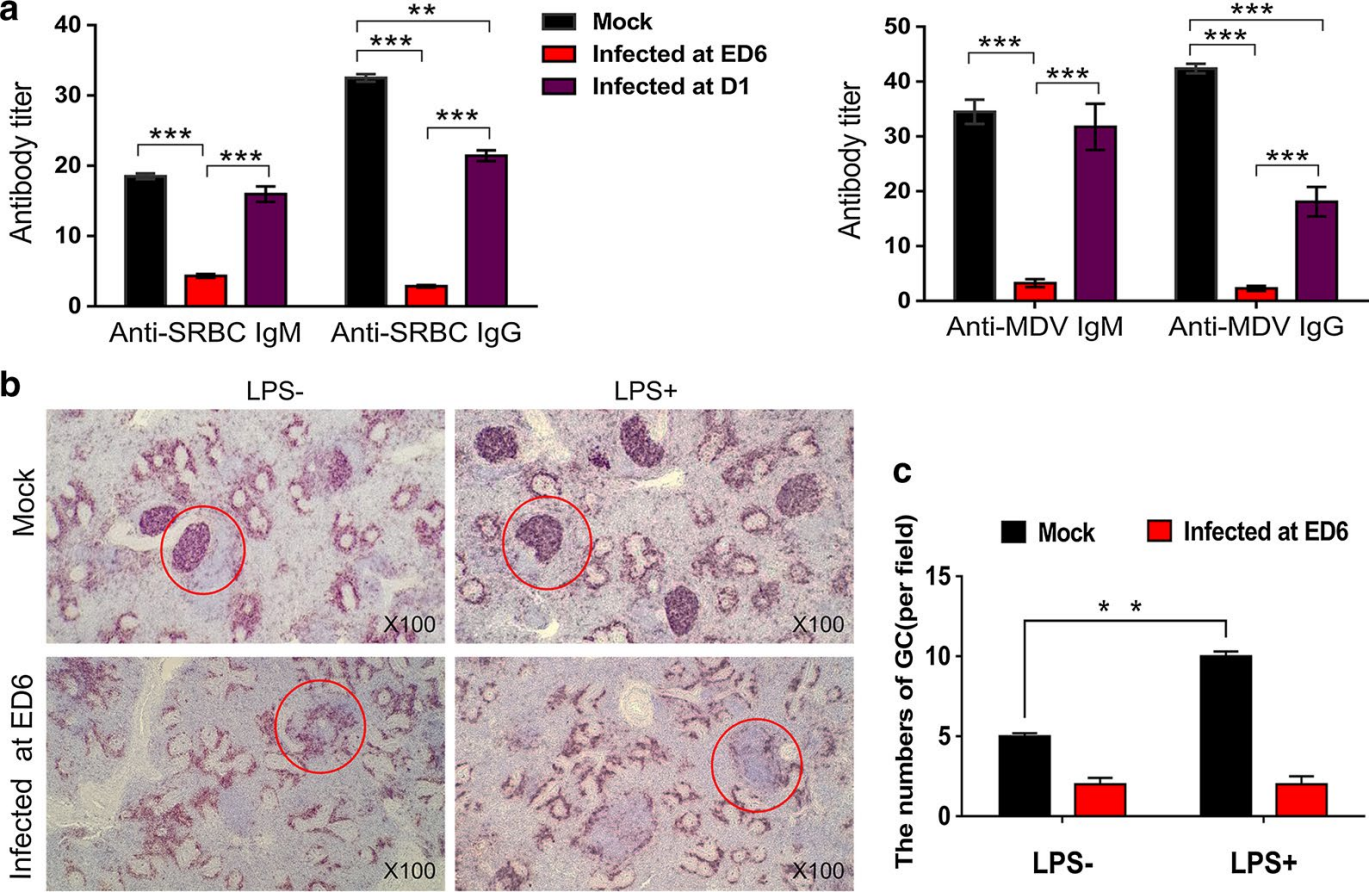
B cell function and responses to antigens and pathogens were impaired in ALV-J congenitally infected chickens.** a** chickens infected by ALV-J at ED 6 showed extremely weakened responses in IgM and IgG classes against both SRBC and MDV, but chickens infected at D 1 had normal values for IgM class Abs. **p < 0.01, ***p < 0.001, two-way ANOVA was performed. **b** IHC staining for chB6 antigen in tissue sections of spleen using mouse anti-chB6 Ab. The numbers of splenic GCs (red circles) and B cells in infected chickens after LPS challenge did not change compared with pre-LPS challenge. However, there were significant differences between the uninfected chickens (n = 6) and infected chickens (n = 6). Sections were stained by BCIP/NBT (black purple) and counterstained by haematoxylin (magnification: ×100). **c** Quantitation of **b**; **p < 0.01, two-way ANOVA was performed

To investigate the function of B cells in chickens infected at ED 6 or D 1, specific antibody titres were detected after the chickens were stimulated with different antigens or pathogens. Current results showed that the immune response capacities of the chickens infected at ED 6 against a thymus-dependent antigen, such as sheep red blood cells (SRBCs), a thymus-independent antigen, such as lipopolysaccharide (LPS), and the Marek’s disease virus (MDV) attenuated serotype 1 vaccine were significantly inhibited compared with the uninfected chickens and posthatch-infected chickens. As shown in Fig. 4a, chickens infected by ALV-J at ED 6 showed extremely weakened responses in IgM and IgG classes against both SRBC and MDV, suggesting early inhibition of B cell development. Interestingly, chickens infected at D 1 had normal values for IgM class Abs, whereas values for IgG class Abs were clearly suppressed, which further indicated the effect of ALV-J on the early development of B cells. Furthermore, we found that the number and volume of splenic GCs markedly increased after LPS challenge in the spleens of uninfected chickens, and the numbers of chB6^+^ cells in the splenic marginal zone and GCs also increased. In contrast to these increases, chickens infected at ED 6 did not have increased numbers of GC and chB6^+^ cells in the spleen after being challenged by LPS (Fig. 4b), and the difference was statistically significant (Fig. 4d).

### ALV-J inhibited the proliferation, maturity, and Ig gene CSR of B cell progenitors in vitro

**Fig. 5:**
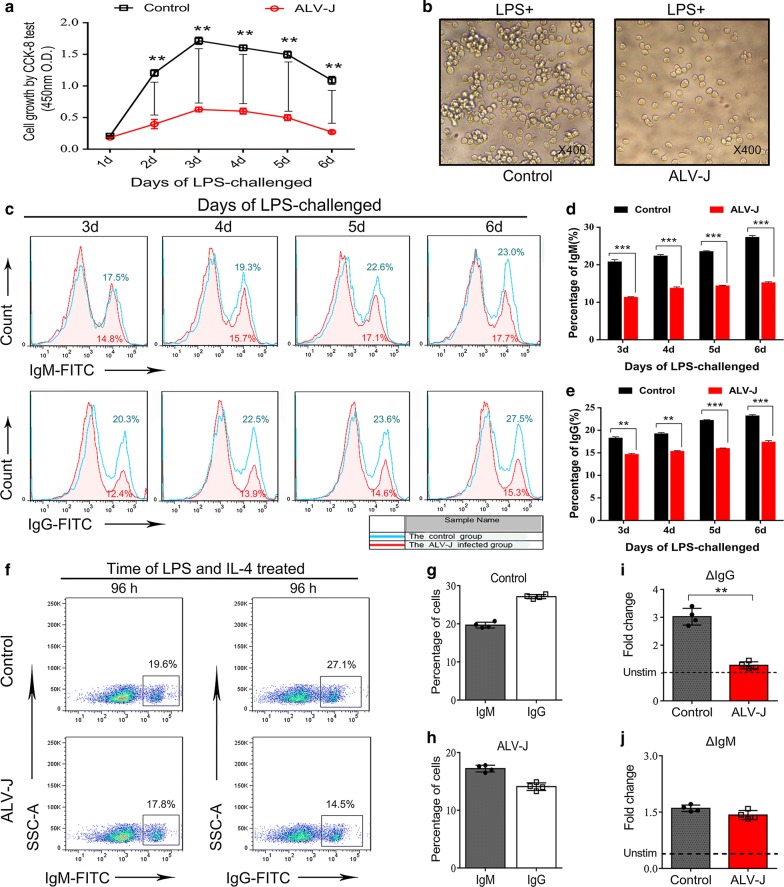
ALV-J inhibited the proliferation, maturity, and Ig gene CSR of B cell progenitors in vitro. **a** CCK-8 test for the mitogen responsiveness of CD117^+^ B cells. Test results showed that B cell proliferation was inhibited by ALV-J. **p < 0.01, two-way ANOVA was performed. **b** Three days after LPS stimulation, there were fewer cell clones in infected groups than in the control group. Moreover, cells in the infected groups were dispersed in the bottom of dish (magnification: ×100). **c** Changes in antibody-producing cells (IgM^+^ and IgG^+^ cells) by flow cytometry analysis. There were fewer IgM^+^ and IgG^+^ cells in the infected groups than in control uninfected groups. **d** and **e** Quantitation of **c**; **p < 0.01, ***p < 0.001, two-way ANOVA was performed to determine the statistical difference. **f** Changes in Ig gene CSR (from IgM to IgG) by flow cytometry analysis. CD117^+^ B cells were cultured with LPS + IL-4 for 4 d, followed by immunofluorescence intracellular staining for Ig genes. **g**, **h** Quantitation of **f**. Fold changes in (**i**) IgG and (**j**) IgM produced by CD117^+^ B cells activated with LPS + IL-4 after 4 d. **p < 0.01, unpaired t-test was performed to determine the statistical difference

The Cell Counting Kit-8 (CCK-8) cell proliferation data showed that the mitogen responsiveness of B cell progenitors was significantly weakened by ALV-J (Fig. 5a). In contrast, the B cell progenitors in the control group could proliferate rapidly upon LPS stimulation. Compared with the control group, the colonies of B cell progenitors infected with ALV-J were few and dispersed (Fig. 5b). To further investigate the effect of ALV-J on the maturity and antibody production capacity of B cell progenitors, surface labelling of IgM and IgG cells was performed for flow cytometry analyses. Consistent with our in vivo findings (as shown in Fig. 2), B cell progenitors infected with ALV-J developed into markedly fewer IgM- and IgG-producing cells in vitro compared with controls (Fig. 5c–e). To directly examine the influence of ALV-J on B cell progenitor activation and CSR, intracellular staining of IgM and IgG was performed for the detection of Ig gene CSR. After the CD117^+^chB6^+^ B cells were cultured and stimulated by LPS and IL-4 for 96 h, the Ig gene CSR was assessed by flow cytometry analyses. The results showed lower IgG expression levels in infected B cells than in the control groups. However, no differences were noted in IgM production in the infected and control groups (Fig. 5f–j). These findings suggested that the maturity of B cell progenitors and Ig gene CSR were interfered by ALV-J in vitro.

## Discussion

As a genus in the family of retroviruses that includes reticuloendotheliosis virus and HIV, ALV-J usually causes haematopoietic cell tumours. However, in the long process of tumour induction, the virus first damages the immune system and causes immunosuppression or immunological tolerance [33, 34]. Regretfully, the potential impact of ALV-J on B cells has been ignored due to fact that the ALV-J mainly targets myeloid lineage cells. Although the pathogenesis of immunological tolerance induced by the virus is complicated, the elimination or inhibition of the activation and proliferation of antigen-specific B and T cells is likely responsible [35]. Consistent with the findings in previous studies, chickens congenitally infected with ALV-J (as mimicked by infection at ED 6 in the current study) were prone to immunological tolerance. Moreover, there were lower values of total IgM and IgG in the blood of chickens infected at ED 6. Here, we demonstrated that the clonal anergy of B cell progenitors induced by ALV-J was associated with immunological tolerance [15, 18, 19].

Combined with previous studies on ALV-J, we considered that ALV-J has a selective effect on the type of lymphocytes and the developmental stage of lymphocytes [36, 37]. Histopathologically, although chicken infection with ALV-J at the embryonic stage and posthatch showed a decrease in thymic medullary lymphocytes, the pathological damage caused by the virus to the thymus was not as significant as the damage caused to the bursa of Fabricius. Therefore, we investigated the relationship between ALV-J and immunological tolerance from the perspective of B cell development and immunological capability. The unique function of B cells in humoral immunity was initially discovered in the bursa of Fabricius [38]. The bursa of Fabricius is the location for Ig gene rearrangement and Ig gene CSR. These crucial findings made it possible to study the effects of ALV-J on B cell development and function. After undergoing productive Ig gene rearrangement and Ig gene CSR, the B cell precursors in the bursa of Fabricius gradually matured and then formed various types of immunoglobulin-producing cells, such as IgM^+^ or IgG^+^ cells [39, 40]. Ig CSR is a natural biological process that alters a B cell’s production of antibodies (such as IgM, IgG, or IgA) from one class to another [41]. In the current study, the data from histopathology and flow cytometry analysis indicated that B cell development was arrested at the early stage. In fact, Ig diversity is generated early in lymphocyte development by the random rearrangement of Ig V (D) J region gene segments. B cells with normal development and function represent the most basic condition for antibody production [42], even if the generation of antigen-specific humoral responses requires the orchestrated coordination of B and T cells. Some studies have suggested that exogenous antigens might induce immunological tolerance of immature B cells in vivo [43]. Our experimental results indicated that the clonal anergy and dysfunction of B cell progenitors induced by ALV-J are important factors for immunological tolerance.

Although there was insufficient evidence to confirm that ALV-J causes B lymphoma, this virus has the capacity to induce lymphocytoma and transform bursal follicles [2, 20, 44]. The previous research results suggested that ALV-J might regulate the genes associated with growth regulation in the bursa of Fabricius [22, 23]. More studies have confirmed that some ALV strains that cause lymphocytic leukaemia, such as RAV-1 and RAV-2, mainly infringe on the B cell precursors in the bursa of Fabricius [45, 46]. These ALV strains blocked the differentiation and emigration of B cells and interfered with the switching of IgM to IgG in B cells. Moreover, the IgM secreted by B lymphoma cells was abnormal [47, 48]. Current data show that the development of B cells and the immune response to specific antigens or pathogen stimulation in infected chickens were severely inhibited when ALV-J infection occurred in early embryonic development. However, the development of B cells in the bursas of Fabricius of posthatch-infected chickens was relatively normal, and the cells produced relatively normal secretory IgM, further confirming that ALV-J affected the early development and function of B cells. As a subgroup of ALVs, it is likely that ALV-J has developed a strategy to affect the development and function of B cell progenitors. Indeed, previous studies have suggested that the specific tolerance against ALV-J infection is due to the expression of envelope glycoproteins with epitopes shared between EAV-HP and ALV-J during embryonic development that would lead to deletion or induction of anergy in ALV-J-responsive immune cells during ontogeny [49–51]. Therefore, these data make us to believe that ALV-J has an important effect on the development of B cell progenitors that leads to the anergy of B cells, which could be associated with immunological tolerance.

## Conclusions

These findings suggested that B cell anergy in congenitally infected chickens is caused by the developmental arrest and dysfunction of B cell progenitors, which is an important factor for the immunological tolerance induced by ALV-J. Together, the results presented here will help us to further understand the pathogenicity of ALV-J and the pathogenesis for immunological tolerance induced by ALV-J. Further studies are needed to investigate the molecular mechanism of ALV-J-induced B cell anergy.

## Methods

### Experimental design

To establish a model of congenital infection of ALV-J, 6-day-old embryos (ED 6) were injected with 100 μL 10^3.8^ TCID_50_ of ALV-J through the allantoic cavity and were incubated in separate incubators. Meanwhile, mock-infected embryos were also incubated. To mimic horizontal infection, the virus was injected into the peritoneal cavity of chicks at one day (D 1) after hatching. The p27 antigen of ALV-J in the cloaca and the total IgM and IgG levels in blood were detected at intervals by ELISA starting at the age of D 2 until the terminal day 28 (D 28). From the age of ED 16 to D 28, the immune organs of chickens (mock, ED 6 infection, and D 1 infection) were sampled every other day and processed for histology (chickens were euthanized with sodium pentobarbital). The development, differentiation, and immunological capability of B cells and are progenitors in vivo and in vitro were assessed by ELISA, IHC, and flow cytometry analysis. All animal experiments described in the present study were approved by the Committee on the Ethics of Animal Experiments of Shandong Province (Permit Number of Protocol: 20160124).

### Materials

Fertilized eggs (n = 320) of Leghorn specific-pathogen-free (SPF) chickens were purchased from the Saisi Company (Jinan, China). ALV-J (NX0101 strain [52]) was maintained in our laboratory. Chickens of mock infection (n = 120), ED6 infection (n = 120) and D1 infection (n = 50) were fed separately in isolated facilities throughout the experiments. Other reagents/materials described in the relevant sections of this article were purchased from commercial companies.

### Histological examination

The bursas of Fabricius, thymes and spleens of chickens (mock, n = 30; ED 6 infection, n = 30; D 1 infection, n = 30) were sampled. These samples were fixed in 4% paraformaldehyde solution and embedded in paraffin wax. Five-micron-thick sections of the middle parts of these immune organs were excised for microscopic examinations. These tissue sections were mounted on triethoxysilane-coated slides before examination with haematoxylin and eosin (H&E) staining. Three randomly selected fields in each target tissue section were photographed and quantified to determine the degree of pathological changes. Meanwhile, consecutive sections of these immune organs were prepared for IHC tests.

### ELISA for p27 antigen, anti-ALV-J antibody, and total IgM or IgG

Wing vein blood and cloaca swab samples were collected from chickens (mock, n = 30; ED 6 infection, n = 30; D 1 infection, n = 30) at the specified intervals (starting at the D 2 until D 28). Blood samples were centrifuged at 800×*g* for 10 min and stored at 4 °C for the detection of anti-ALV-J Ab and total IgM and IgG. Anti-ALV-J Ab or p27 antigen was detected using a commercial ELISA test kit (IDEXX USA Inc., Beijing, China) according to the manufacturer’s instruction. The levels of p27 antigen of ALV-J or anti-ALV-J Ab were evaluated by calculating the s/p ratio. The value of the cut-off was 0.2 (s/p ratio), as recommended by the manufacturer. Moreover, the total IgM and IgG levels in blood were tested using commercial ELISA test kits (Abcam, Cambridge, USA). In the above tests, each biological sample was tested in triplicate. The p27 antigen-positive chickens were euthanized on the day of detection, and their organs were sampled and preserved for the next tests.

### Immunohistochemistry

IHC was performed to detect the expression levels of chB6, IgM, IgG, and ALV-J antigen in tissues according to the instructions for the DouMaxVision™ kits (Maixin-Bio Ltd., Fuzhou, China). Primary antibodies include mouse anti-chicken chB6 monoclonal antibodies (mAb) (1:200; Southern Biotech, Birmingham, USA), rabbit anti-chicken IgM mAb (1:800; Abcam, Massachusetts, USA), rabbit anti-chicken IgG mAb (1:800; Jackson, Westgrove, PA), and rabbit anti-chicken ALV-J polyclonal Ab (1:200; made in our laboratory). Secondary antibodies include alkaline phosphatase-labelled goat anti-mouse IgG polymer and horseradish peroxidase-labelled goat anti-rabbit IgG polymer. Briefly, after the antigen was retrieved and blocked with 10% normal goat serum, each tissue section was incubated with primary antibody for 1 h at room temperature, after which the section was washed with PBS three times and incubated with secondary antibody for 15 min at 37 °C. Sections were stained by 5-bromo-4-chloro-3-indolyl-phosphate/nitro blue tetrazolium (BCIP/NBT), 3-amino-9-ethylcarbozole (AEC) or 3, 3′-diaminobenzidine (DAB) after rinsing, counterstained by haematoxylin and sealed by an aqueous mounting media. Negative controls were also performed with the same tissues. Six randomly selected fields of positive expression in each target tissue section were photographed and analysed in Image J software to accurately calculate the positive area and to measure the mean optical density.

### Flow cytometry analysis for the differentiation of B cell progenitors and the percentage of ALV-J-infected B cells

At the ages of ED 20, D 4, and D 8, erythrocyte-depleted bursal cells from congenitally infected chickens (n = 5 per point of time) and mock-infected chickens (n = 5 per time point) were suspended in cold PBS and stained with mouse anti chicken chB6-FITC mAb and mouse anti chicken CD117-PE mAb (Southern Biotech, Birmingham, USA) for flow cytometry analysis. In addition, erythrocyte-depleted bursal cells from 14-days-old chickens, including mock-infected chickens (n = 10), chickens infected at ED 6 (n = 10), and chickens infected at D 1 (n = 10), were sampled and analysed by flow cytometry for the percentage of ALV-J-infected B cells. Before flow cytometry analysis, these cells were stained with mouse anti-chicken chB6-FITC mAb (Southern Biotech, Birmingham, USA) and PE-labelled ALV-J Ab (PE was purchased from Expedeon Company in the UK, the ALV-J Ab was made in our laboratory). In these tests, mouse IgG1κ-FITC and mouse IgG2a-PE isotype antibodies (Southern Biotech, Birmingham, USA) were also used. Cells were analysed by a BD FACS Aria II instrument (BD Biosciences). Data were analysed using FlowJo (TreeStar) software.

### Stimulation by antigen and antibody determination

In this test, the immune responses of chickens against SRBC (Solarbio, China), attenuated MDV serotype 1 vaccine (Harbin Veterinary Medicine Research Institute, china), and LPS (O55:B55; Solarbio, China) were assessed. In brief, 0.5 mL of 6 × 10^7^ SRBC and 0.2 mL of attenuated MDV vaccine were injected into chickens (mock, n = 10; ED6 infection, n = 10; D1 infection, n = 10) through the abdominal cavity and via subcutaneous injection, respectively, at 1, 2, and 2.5 weeks of age. Heparinized blood was sampled by heart puncture 4 days after the second immunization and 7 days after the final immunization. The titres of IgM and IgG-class Abs against SRBC and MDV were tested by ELISA according to the instructions from the commercial ELISA test kit (Chicken anti-MDV Ab (IgM/IgG) ELISA test kit and Chicken anti-SRBC Ab (IgM/IgG) ELISA test kit, Berseebio, China). Furthermore, to investigate the effect of ALV-J on the proliferation capacity of B cells in vivo, both the mock-infected chickens (n = 6) and the congenitally infected chickens (n = 6) were injected with LPS (40 mg/kg) through the abdominal cavity at the age of D 16. After four days, the spleens were collected and processed for IHC. B cells in the spleen were stained with mouse anti-chB6 Ab. Six randomly selected fields of the positive expression in each target tissue section were photographed to accurately calculate the numbers of GCs.

### Sorting and culture of B cell progenitors

Erythrocyte-depleted bursal cells from normal chickens (n = 30) on ED20 were labelled with mouse anti-chicken chB6-FITC mAb (Southern Biotech, Birmingham, USA) and mouse anti-chicken CD117-PE mAb (Southern Biotech, Birmingham, USA) and were then sorted by fluorescence activated cell sorting (FACS) according to a modified protocol in a sterile operation [53]. Briefly, for each round of sorting, 1 × 10^6^ bursal cells were labelled with Fixable Aqua Dead Cell Stain (Life Technologies) for the detection of dead cells and stained with 2 μg/mL chB6-FITC mAb and CD117-PE mAb for 30 min at 4 °C. Single cells were sorted by a BD FACS Aria II instrument (BD Biosciences). Meanwhile, mouse IgG_1_κ-FITC/PE isotype antibody (Southern Biotech, Birmingham, USA) was also used to establish the assay’s validity. The purity of the sorted cells was ≥ 98%, as analysed with FACSDiva software (BD Biosciences). Then, these CD117^+^chB6^+^ cells were seeded in 24-well plates at 1 × 10^6^ cells/mL and maintained at 37 °C with 5% CO_2_ in a culture medium containing IMDM (Gibco, California, USA), 20% chicken serum (Gibco, California, USA), 50 ng/mL chicken stem cell growth factor (SCF) and 20 ng/mL chicken interleukin-7 (IL-7) (Creative Biomart, New York, USA), 100 U/mL penicillin, and 100 μg/mL streptomycin.

### Mitogenic assays and detection of Ig gene CSR

The CD117^+^chB6^+^ cells were infected by ALV-J and expanded through stimulation with 40 µg/mL lipopolysaccharide (LPS) (O55:B55; Solarbio, China). Culture medium with LPS was half-quantity changed every two days and added to the new medium. Cells were harvested every day for the Cell Counting Kit-8 (CCK-8) (Solarbio, China) test and flow cytometry analysis of IgM^+^ and IgG^+^ cells. The CCK-8 test was performed according to the manufacturer’s protocol. Mouse anti-chicken IgM-FITC mAb (Abcam, Massachusetts, USA) and mouse anti-chicken IgG-FITC mAb (Jackson, Westgrove, PA) were used for the surface labelling of IgM^+^ and IgG^+^ cells. Cells were stained with antibodies for 30 min at 4 °C and were then immediately analysed on a BD FACS Aria II instrument after washing. Data were analysed using FlowJo (TreeStar) software.

To detect the Ig gene class-switch of the B cell progenitor response to mitogen in vitro, the CD117^+^chB6^+^ cells were plated at 1 × 10^6^ cells/mL in 24-well plates and cultured with IMDM supplemented with 20% chicken serum, 50 ng/mL chicken SCF, 20 ng/mL chicken IL-7, 100 U/mL penicillin, and 100 μg/mL streptomycin. Cells were then stimulated with 40 µg/mL LPS and 20 ng/mL IL-4. After 96 h, cells were harvested and fixed in 2% paraformaldehyde solution. IgM-FITC mAb and IgG-FITC mAb were used for the intracellular staining of IgM and IgG. The flow cytometry analysis was performed as described above.

### Statistical analyses

Multiple sets of data comparisons were measured using two-way analysis of variance (ANOVA). The unpaired *t* test was used when two groups were compared. The results were accepted as significantly different when *p* ≤ 0.05, *p* ≤ 0.01, or *p* ≤ 0.001. Analysis and plotting of data were performed using GraphPad Prism 6.0 and are expressed as the mean ± SEM.
